# Error-Robust Distributed Denial of Service Attack Detection Based on an Average Common Feature Extraction Technique

**DOI:** 10.3390/s20205845

**Published:** 2020-10-16

**Authors:** João Paulo Abreu Maranhão, João Paulo Carvalho Lustosa da Costa, Edison Pignaton de Freitas, Elnaz Javidi, Rafael Timóteo de Sousa Júnior

**Affiliations:** 1 Department of Electrical Engineering, University of Brasília, Brasília 70910-900, Brazil; joaopaulo.dacosta@ene.unb.br (J.P.C.L.d.C.); desousa@unb.br (R.T.d.S.J.); 2 Department 2-Campus Lippstadt, Hamm-Lippstadt University of Applied Sciences, 59063 Hamm, Germany; 3 Informatics Institute, Federal University of Rio Grande do Sul, Porto Alegre 91509-900, Brazil; epfreitas@inf.ufrgs.br; 4 Department of Mechanical Engineering, University of Brasília, Brasília 70910-900, Brazil; elnaz.javidi@gmail.com

**Keywords:** cyber–physical systems, machine learning, tensor decomposition, classification, error-robustness

## Abstract

In recent years, advanced threats against Cyber–Physical Systems (CPSs), such as Distributed Denial of Service (DDoS) attacks, are increasing. Furthermore, traditional machine learning-based intrusion detection systems (IDSs) often fail to efficiently detect such attacks when corrupted datasets are used for IDS training. To face these challenges, this paper proposes a novel error-robust multidimensional technique for DDoS attack detection. By applying the well-known Higher Order Singular Value Decomposition (HOSVD), initially, the average value of the common features among instances is filtered out from the dataset. Next, the filtered data are forwarded to machine learning classification algorithms in which traffic information is classified as a legitimate or a DDoS attack. In terms of results, the proposed scheme outperforms traditional low-rank approximation techniques, presenting an accuracy of 98.94%, detection rate of 97.70% and false alarm rate of 4.35% for a dataset corruption level of 30% with a random forest algorithm applied for classification. In addition, for error-free conditions, it is found that the proposed approach outperforms other related works, showing accuracy, detection rate and false alarm rate of 99.87%, 99.86% and 0.16%, respectively, for the gradient boosting classifier.

## 1. Introduction

Cyber–Physical Systems (CPSs) consist of a set of networked components including sensors, control processing units and communication devices applied to the monitoring and management of physical infrastructures [1]. CPSs are typically used for safety-critical applications, such as in avionics, instrumentation, defense systems and critical infrastructure control, for instance, electric power, water resources and communications systems [2]. Consequently, potential cyber and physical attacks can lead to information leakage, extensive economic damage and critical infrastructure destruction [3].

A CPS architecture is typically composed of five layers, namely, physical layer, sensor/actuator layer, network layer, control layer, and information layer. The physical layer consists of the physical objects or processes monitored by CPSs. In addition, the sensor/actuator layer is composed of sensors, which measure data obtained from the physical layer, and by actuators, which execute specific actions under the control of the above layers. For example, in the air traffic control, sensors receive measurement data collected from a sensor array-based localization system, whereas actuators are used to neutralize unmanned aerial vehicles detected within the controlled airspace [4]. Additionally, the network layer is responsible for network sensors and actuators, as well as connecting the sensor/actuator and the control layers through communication devices and protocols. Furthermore, the control layer, through intelligent electronic devices, programmable logic controllers and remote terminal units, is responsible for the locally distributed control action level. Such a layer forwards the measurement data to human operators in the information layer, which monitor the system and take actions whenever required [1].

In this sense, it is crucial to develop highly reliable intrusion detection systems for CPSs such that safety-critical applications can be controlled and protected in an efficient way. Currently, intrusion detection schemes are highly sophisticated, involving advanced signal processing techniques [5], as well as machine learning (ML)-based solutions [6]. The scope of this paper is the security of the CPS against Distributed Denial of Service (DDoS) attacks, which are one of the major security threats in existence today. DDoS attacks are launched by thousands of compromised machines, called “zombies”, which together establish a “zombie” network. Such zombies perform massive attacks against a victim, depleting its bandwidth and network resources. Common DDoS detection models include the traffic entropy model and history-based Internet Protocol (IP) filtering. However, with the development of cloud computing, Internet of Things (IoT) and artificial intelligence techniques, such traditional network intrusion detection solutions cannot face modern DDoS attack strategies, which are harder to detect and prevent [7].

In order to obtain higher performance, ML-based intrusion detection systems (IDSs) must be trained with massive amounts of data. Usually, large datasets have inherent multidimensional structure, which can be better explored by applying tensor signal processing techniques. However, a potential drawback consists of the presence of errors in such large datasets. In this case, such errors can refer to uncalibrated measures that occurred during the process of dataset creation [8], or due to false data injection performed by attackers on publicly available datasets [9], leading to data corruption. Such a fact can degrade the performance of the ML classifier and, consequently, reduce the reliability of the DDoS attack detection model.

To face the above-mentioned issues, we propose an error-robust tensor-based technique for DDoS attack detection. First, we filter out, from the dataset, the average value of the common features among instances such that the machine learning classification algorithms can benefit from the more discriminative individual information at each instance during the training phase. In this paper, decision tree (DT), random forest (RF) and gradient boosting (GB) classifiers are applied for performance evaluation, whereas the CICDDoS2019 and CICIDS2017 datasets are considered in numerical simulations. According to the results in Section 6, the proposed scheme outperforms the well-known Higher-Order Orthogonal Iteration (HOOI) and Higher-Order Singular Value Decomposition (HOSVD) techniques in terms of accuracy, detection rate, false alarm rate, area under the precision–recall curve and Matthews correlation coefficient.

The main research contributions of this paper are summarized as follows:

- The proposal of a novel technique in which the average value of the common features among instances is filtered out from the dataset by applying the HOSVD low-rank approximation scheme, improving the performance of the intrusion detection system.
- The comparison with different state-of-the-art low-rank approximation techniques in order to show the higher performance and error-robustness of the proposed approach.

The remainder of this paper is organized as follows. Section 2 presents the related works. Section 3 introduces the data model. In Section 4, the theoretical background is introduced. Section 5 shows the proposed tensor based scheme for DDoS attack detection in CPSs. In Section 6, simulation results are presented and discussed. Section 7 draws the conclusions.

## 2. Related Works

In this section, the related works are presented and discussed. Since the proposed scheme is based on multidimensional signal processing techniques applied on DDoS attack detection, we discuss papers related to multilinear algebra and distributed denial of service detection systems. In [10,11], the authors presented multidimensional solutions for image classification. However, whereas the former proposed common and individual feature extraction techniques based on LL1 decomposition, the latter applied the HOSVD algorithm for classifying corrupted images. In addition, Lathauwer et al. [12] proposed the classical HOOI low-rank approximation technique, widely applied for tensor denoising. In [5], the authors proposed a signal processing-based approach in which model order selection and eigen similarity analysis are applied for detecting and identifying the time instants and ports exploited by attackers. Finally, specifically regarding DDoS attack detection, three researches can be cited. Hosseini and Azizi [13] proposed a hybrid framework based on a data stream approach for DDoS attack detection where the computational load is divided between the client and proxy side. Next, Lima Filho et al. [14] proposed a random forest-based DDoS detection system in which several volumetric attacks, such as Transmission Control Protocol (TCP) flood, User Datagram Protocol (UDP) flood, and Hyper Text Transfer Protocol (HTTP) flood, are early identified. Finally, Wang et al. [6] proposed a method for detecting DDoS attacks in which the optimal features are obtained by combining feature selection and multilayer perceptron (MLP) classification algorithm. Further, when considerable detection errors are dynamically perceived, a feedback mechanism reconstructs the IDS.

In Table 1, we summarize the general aspects of the above mentioned related works, highlighting their aims, proposed solutions, pros and cons.

## 3. Data Model

This section presents the data model adopted in this paper and is divided into two subsections. First, Section 3.1 shows the mathematical notation used throughout this paper. Next, a brief description of the data modeling is presented in Section 3.2.

### 3.1. Mathematical Notation

In this subsection, we present the mathematical notation used throughout this paper. Italic letters (a,b,c,A,B,C) represent scalars, lowercase bold letters represent column vectors (a,b,c) and uppercase bold letters represent matrices (A,B,C). Higher order tensors are denoted by uppercase bold calligraphic letters (A,B,C). The concatenation of the tensors A and B along the *r*-th dimension is defined as ${\left[A\;\;|\;\;B\right]}_{r}$. Transposition and Hermiticity of a matrix are represented by the superscripts ${\left\{\cdot \right\}}^{T}$ and ${\left\{\cdot \right\}}^{H}$, respectively. The operator diag(·) transforms its argument vector into the main diagonal of a diagonal matrix. The Hadamard product is represented by operator ⊙.

Furthermore, the *r*-th mode unfolding of the tensor X is denoted as ${\left[X\right]}_{\left(r\right)}$, which is obtained by varying the *r*-th index along the rows and stacking all other indices along its columns. Additionally, $Y=X\times_{r}B$ denotes the *r*-mode product between the tensor X and the matrix B. In a matricized fashion, such a product can be expressed as ${\left[Y\right]}_{\left(r\right)}=B{\left[X\right]}_{\left(r\right)}$.

### 3.2. Data Modeling

In this paper, the dataset matrix $X\in R^{M\times N}$ is modeled in the following fashion:

(1) $$X=X_{0}+N$$

where $X_{0}\in R^{M\times N}$ is the error-free dataset matrix, $N\in R^{M\times N}$ is the error matrix, *M* is the number of instances and *N* is the number of features. The matrix N represents generalized perturbations added to $X_{0}$, for instance, false data injection attacks, which are commonly used to fool machine learning classifiers. The *m*-th instance and the *n*-th feature are, respectively, given by $X_{m,:}$ for m=1,…,M and $X_{:,n}$ for n=1,…,N. The class label vector is denoted by $y={\left[y_{1},\ldots ,y_{M}\right]}^{T}\in R^{M}$, where $y_{m}$ indicates if the *m*-th instance $X_{m,:}$ for m=1,…,M is legitimate traffic or DDoS attack.

Furthermore, we can rewrite the dataset matrix X in (1) in a tensor form. Initially, each instance $X_{m,:}\in R^{N}$ for m=1,…,M is reshaped as a tensor with dimensions $N_{1}\times \cdots \times N_{R}$, such that $N=\prod_{r=1}^{R}N_{r}$. Then, the *M* tensors are stacked along the (R+1)-th dimension, generating the dataset $X\in R^{N_{1}\times \cdots \times N_{R}\times M}$ denoted as:

(2) $$X=X_{0}+N$$

where $X_{0}\in R^{N_{1}\times \cdots \times N_{R}\times M}$ is the error-free dataset tensor and $N\in R^{N_{1}\times \cdots \times N_{R}\times M}$ is the error tensor. The *r*-th mode unfolding matrix of X is given by ${\left[X\right]}_{\left(r\right)}\in R^{N_{r}\times \prod_{j\neq r}N_{j}\times M}$. Note that the dataset matrix $X\in R^{M\times N}$ in (1) corresponds to the (R+1)-th unfolding matrix ${\left[X\right]}_{\left(R+1\right)}\in R^{M\times \prod_{r=1}^{R}N_{r}}$.

## 4. Theoretical Background

This section presents the theoretical background and is divided into two subsections. First, Section 4.1 introduces the taxonomy of DDoS attacks. Next, Section 4.2 details the DDoS attack datasets adopted in this paper.

### 4.1. Taxonomy of DDoS Attacks

Distributed Denial of Service attacks are one of the most important security threats nowadays. In a DDoS attack, a large volume of traffic is sent through the network, exhausting the network resources, as well as the overall bandwidth and individual node resources [15]. Consequently, the victim is forced to slow down, crash or shut down due to multiple connection requests during a period of time [16].

Since networks and servers became more robust in identifying network layer DDoS attacks, hackers responded by moving up the OSI model stack to higher layers [17]. For instance, several DDoS attacks exploit vulnerabilities present in the application layer, reproducing the behavior of legitimate customers and, consequently, are not detected by most of the conventional IDSs [18]. In this sense, currently, several researches in the literature broadly classify DDoS attacks into three types: application-layer attacks, resource exhaustion attacks, and volumetric attacks [19], which are described as follows:

- Application-Layer Attack: in this type of attack, vulnerabilities present in the application are used by an attacker, making it inaccessible by legitimate users [19]. Instead of depleting the network bandwidth, the server resources, such as CPU, database, socket connections or memory, are exhausted by application-layer DDoS attacks. In addition, such attacks present some subtleties which make them harder to detect and mitigate: they are performed through legitimate HTTP packets, with a low traffic volume, presenting high resemblance to flash crowds [17]. HTTP and Domain Name System (DNS)-based DDoS attacks are examples of application-layer attacks.
- Resource Exhaustion Attack: In this category, hardware resources of servers, such as memory, CPU, and storage, are depleted. Consequently, they become unavailable for legitimate accesses. Resource exhaustion attacks are also known as protocol-based attacks, since vulnerabilities in protocols are exploited. For example, in an SYN flood attack, a hacker exploits the TCP three-way handshake process. After receiving a high volume of SYN packets, the targeted server responds with SYN/ACK packets and leaves open ports to receive the final ACK packets, which never arrive. This process continues until all ports of the server are unavailable.
- Volumetric Attack: In this type of attack, the bandwidth of the target system is exhausted by a massive amount of traffic. Since such attacks are launched by using amplification and reflection techniques, they are considered as the simplest DDoS attacks to be employed [18]. UDP flood and Internet Control Message Protocol (ICMP) flood can be cited as volumetric attacks.

### 4.2. CICDDoS2019 and CICIDS2017 Datasets

In this paper, we consider two datasets provided by the Canadian Institute of Cybersecurity (CIC) for network intrusion detection models, namely, CICDDoS2019 [20] and CICDIS2017 [21]. CICDDoS2019 is a novel benchmark dataset composed by several network traffic features, with millions of labeled legitimate and DDoS attack instances [22]. The dataset was generated in two distinct days. In 12 January 2019, the training set was captured, containing 12 different types of DDoS attacks, namely, DNS, WebDDoS, LDAP, MSSQL, NetBIOS, NTP, SNMP, SSDP, UDP, SYN, TFTP and UDP-Lag based attacks. Next, in 11 March 2019, the testing set was generated, with seven DDoS attack types, including LDAP, MSSQL, NetBIOS, UDP, SYN and UDP-Lag based attacks, plus Port Scan. All DDoS attacks were separated in different PCAP files, according to their types.

Similarly to CICDDoS2019, CICIDS2017 is a completely labeled dataset that contains legitimate traffic and the most up-to-date common network attacks. The dataset was generated in five days, from Monday, 3 July 2017, to 7 July 2017, and is publicly available in PCAP and CSV files. On Monday, only legitimate traffic was captured, whereas different types of network attacks were captured in the following days. The malicious activities include common updated attacks, for example, DDoS, Denial of Service (DoS), Brute Force, Cross-Site Scripting (XSS), SQL Injection, Infiltration, Port Scan and Botnet [23]. Particularly, DDoS attacks were generated on 7 July 2017. Since we focus on DDoS attack detection, only legitimate and DDoS attack instances present in the traces of 3 July 2017, and 7 July 2017, respectively, are used in this research.

## 5. Proposed Average Common Feature Extraction Technique for DDoS Attack Detection in Cyber–Physical Systems

This section presents the proposed average common feature extraction scheme for DDoS attack detection in CPSs. First, we introduce the concept of common and individual features of a given dataset. Such concept is well-known in image classification problems, in which data share some common variables while exhibiting their own features simultaneously [24]. Let us assume a tensor $Y\in R^{I_{1}\times I_{2}\times S}$ composed of the slices $Y_{:,:,s}$ for s=1,…,S. Each frontal slice $Y_{:,:,s}$ is equivalent to a combination of the three base colors, namely, green, red and blue, represented by the matrices $B_{G}\in R^{I_{1}\times I_{2}}$, $B_{R}\in R^{I_{1}\times I_{2}}$ and $B_{B}\in R^{I_{1}\times I_{2}}$. Usually, the base colors are obtained through tensor decompositions, such as the LL1 decomposition with non-negativity constraint, such that $Y=\left(B_{G}\times_{3}c_{G}\right)+\left(B_{R}\times_{3}c_{R}\right)+\left(B_{B}\times_{3}c_{B}\right)$, where $c_{G}\in R^{S}$, $c_{R}\in R^{S}$ and $c_{B}\in R^{S}$ contain the intensity values of the red, green and blue colors, respectively [10]. Note that Y presents rank three, which corresponds to the number of base colors. Alternatively, the base colors can be stacked along the 3rd dimension, generating $B\in R^{I_{1}\times I_{2}\times 3}$, whereas the vectors $c_{G}$, $c_{R}$ and $c_{B}$ can be grouped into the matrix $C\in R^{S\times 3}$. The tensor B, known as the common feature tensor, can also be represented as $\tilde{Y}$, as a reference to the original dataset Y.

After extracting the common features, only the more discriminative individual information at each instance is used during the training phase, which improves the performance of the machine learning classifier [10]. In this sense, due to the considerable results for image classification, the concept of common and individual feature extraction shows an outstanding potential for detecting network intrusion by using large datasets in ML classifier training. Hence, a similar procedure is adopted in this paper, such that the average value of the common features among dataset instances is filtered out from the data. As a consequence, the ML classifier takes advantage of the benefits from the resulting filtered dataset. In order to improve the readability, the mathematical symbols used throughout this section are summarized in Table 2.

Before applying the feature extraction technique on the dataset tensor, three steps are necessary, namely, dataset splitting, dataset pre-processing and multilinear rank estimation, which are described as follows.

- Dataset Splitting: First, the DDoS attack dataset $X\in R^{N_{1}\times \cdots \times N_{R}\times M}$ is split into the training and testing tensors $X^{\mathrm{tr}}\in R^{N_{1}\times \cdots \times N_{R}\times M^{\mathrm{tr}}}$ and $X^{\mathrm{te}}\in R^{N_{1}\times \cdots \times N_{R}\times M^{\mathrm{te}}}$, where $M^{\mathrm{tr}}$ and $M^{\mathrm{te}}$ are the number of training and testing instances, respectively, with $M=M^{\mathrm{tr}}+M^{\mathrm{te}}$.
- Dataset Pre-Processing: The training and testing datasets, $X^{\mathrm{tr}}$ and $X^{\mathrm{te}}$, are submitted to a preprocessing step, which includes data cleansing, feature scaling and label encoding. Initially, several rows containing missing values (NaN) and infinity values (Inf) are removed from the dataset. Next, all features are normalized to the range [0−1] such that features with a higher order of magnitude do not dominate lower variables. Then, since we are dealing with binary classification, legitimate and DDoS attack instances are labeled as 0 and 1, respectively.
- Multilinear Rank Estimation: Finally, we estimate the multilinear ranks $\left(d_{1}^{\mathrm{tr}},\ldots ,d_{R+1}^{\mathrm{tr}}\right)$ and $\left(d_{1}^{\mathrm{te}},\ldots ,d_{R+1}^{\mathrm{te}}\right)$ corresponding to the tensors $X^{\mathrm{tr}}$ and $X^{\mathrm{te}}$, respectively. The parameters $d_{r}^{\mathrm{tr}}$ and $d_{r}^{\mathrm{te}}$ for r=1,…,R+1 are estimated by using multidimensional model order selection (MOS) schemes, such as the *R*-D Minimum Description Length [25].

**Figure 1:**
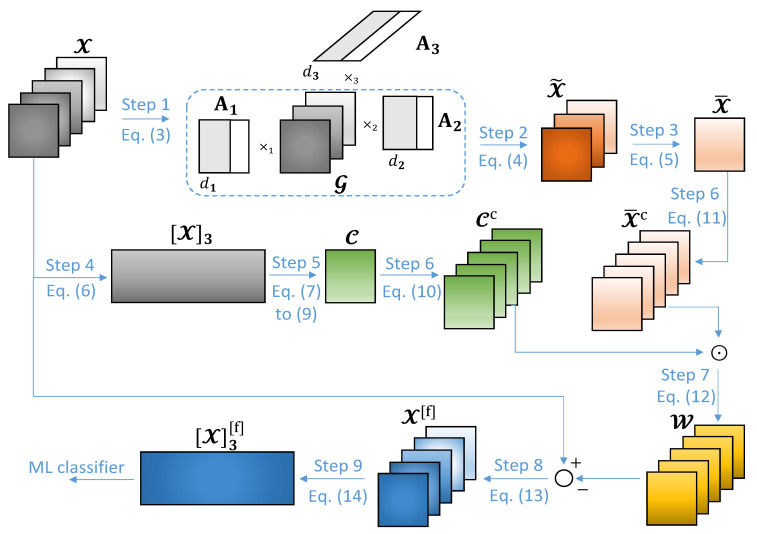
Proposed average common feature extraction technique for DDoS attack detection in Cyber–Physical Systems (CPSs). For simplicity, we depict the filtering process of a three-dimensional dataset tensor $X\in R^{N_{1}\times N_{2}\times M}$.

After the above-mentioned steps, $X^{\mathrm{tr}}$ is forwarded to the proposed average common feature extraction technique for DDoS attack detection, such that the training phase is initialized. Next, when the training process is finished, $X^{\mathrm{te}}$ is sent to the trained IDS for classification. For simplicity, from this point on, $X\in R^{N_{1}\times \cdots \times N_{R}\times M}$ can refer to the training or testing dataset tensors. The steps of the proposed scheme, shown in Figure 1, are discussed as follows.

- Step 1: Computing the HOSVD of X.
  In Step 1 of Figure 1, we compute the Higher-Order Singular Value Decomposition (HOSVD) of the dataset tensor $X\in R^{N_{1}\times \cdots \times N_{R}\times M}$. Here, we intend to obtain the core tensor, $G\in R^{d_{1}\times \cdots \times d_{R+1}}$, as well as the first *R* factor matrices, $A_{r}\in R^{N_{r}\times d_{r}}$ for r=1,…,R, where $\left(d_{1},\ldots ,d_{R+1}\right)$ is the multilinear rank of X. Such tensors are used in Step 2 to compute the common feature tensor, $\tilde{X}\in R^{N_{1}\times \cdots N_{R}\times d_{R+1}}$.
  The HOSVD of X is given by:
  (3) $$X=G\times_{1}A_{1}\cdots \times_{R}A_{R}\times_{R+1}A_{R+1}$$
  Usually, the number of common features among the dataset instances is obtained empirically. However, a considerable performance is achieved by considering $d_{R+1}$ as an estimate of the number of common features, as shown in the simulations of Section 6. We refer here to [25] to estimate the number of common features.
- Step 2: Computing the common feature tensor, $\tilde{X}$.
  In Step 2 of Figure 1, we compute $\tilde{X}\in R^{N_{1}\times \cdots N_{R}\times d_{R+1}}$, which contains the common features among the dataset instances $X_{:,\ldots ,m}\in R^{N_{1}\times \cdots \times N_{R}}$ for m=1,…,M. The tensor $\tilde{X}$ is defined as the *r*-mode product between the core tensor G and the first *R* factor matrices [26],
  (4) $$\tilde{X}=G\times_{1}A_{1}\cdots \times_{R}A_{R}$$
- Step 3: Computing the average common feature tensor, $\bar{X}$.
  Next, in Step 3 of Figure 1, we compute $\bar{X}\in R^{N_{1}\times \cdots \times N_{R}}$, which corresponds to $\tilde{X}$ averaged along the (R+1)-th dimension, i.e.,
  (5) $$\bar{X}=\frac{1}{d_{R+1}}\sum_{d=1}^{d_{R+1}}{\tilde{X}}_{:,\ldots ,d}$$
- Step 4: Obtaining the (R+1)-th mode unfolding matrix, ${\left[X\right]}_{\left(R+1\right)}$.
  Following, in Step 4 of Figure 1, we obtain the (R+1)-th mode unfolding matrix of X, given by ${\left[X\right]}_{\left(R+1\right)}\in R^{M\times N}$. In general, the *r*-th unfolding matrix ${\left[X\right]}_{r}$ is obtained after each element $\left(x_{1},\ldots ,x_{R+1}\right)$ in X is mapped to the element $\left(x_{r},j\right)$ in ${\left[X\right]}_{r}$ as follows:
  (6) $$j=1+\sum_{\begin{array}{c} k=1\\ k\neq r \end{array}}^{R+1}\left(x_{k}-1\right)J_{k},\;\;\mathrm{with}\;\;J_{k}=\prod_{\begin{array}{c} m=1\\ m\neq r \end{array}}^{k-1}N_{m}$$
  Such a matrix is used in Step 5 in order to compute the weights to be applied on $\bar{X}$ for dataset filtering.
- Step 5: Computing the weight tensor, C.
  In Step 5 of Figure 1, we compute the weight tensor $C\in R^{N_{1}\times \cdots \times N_{R}\times M}$, which is used for dataset filtering in Step 7. First, the covariance matrix $R_{\mathrm{xx}}\in R^{N\times N}$ of the (R+1)-th mode unfolding matrix ${\left[X\right]}_{\left(R+1\right)}\in R^{M\times N}$, as well as its eigenvalue decomposition, are obtained as follows:
  (7) $$R_{\mathrm{xx}}=\frac{1}{M}{\left[X\right]}_{\left(R+1\right)}^{H}{\left[X\right]}_{\left(R+1\right)}$$
  (8) $$R_{\mathrm{xx}}=E\Lambda E^{H}$$
  where $E\in R^{N\times N}$ is the eigenvector matrix of $R_{\mathrm{xx}}$ and $\Lambda \in R^{N\times N}$ contains the eigenvalues $\lambda_{1},\ldots ,\lambda_{N}$ of $R_{\mathrm{xx}}$ in its diagonal. Such eigenvalues are sorted in descending order so that $\lambda_{1}$ is the largest one.
  Before subtracting the average common features from X, we have to multiply each one of the elements of $\bar{X}$ by a positive number smaller than 1. This can be done by computing the Hadamard product between $\bar{X}$ and a weight tensor $C\in R^{N_{1}\times \cdots \times N_{R}\times M}$. The tensor C can be obtained empirically or by some adaptive technique such that the errors between the expected and predicted classifications during the training phase of a ML classifier are minimized. In this paper, we adopt the following empirical approximation: all elements of C are equal to the average eigenvalue $\bar{\lambda }$ of $R_{\mathrm{xx}}$, i.e.,
  (9) $$\bar{\lambda }=\sum_{n=1}^{N}\lambda_{n}$$
  where $\lambda_{n}$ for n=1,…,N are the eigenvalues of $R_{\mathrm{xx}}$.
- Step 6: Obtain the concatenated tensors, $C^{C}$ and ${\bar{X}}^{C}$.
  In Step 6 of Figure 1, *M* copies of C are concatenated along the (R+1)-th dimension, generating the tensor $C^{C}\in R^{N_{1}\times \cdots \times N_{R}\times M}$. The same procedure is adopted for $\bar{X}$ in order to obtain ${\bar{X}}^{C}\in R^{N_{1}\times \cdots \times N_{R}\times M}$. Both computations can be expressed as L:
  (10) $$C^{C}={\left[C\;\;|\;\;\ldots \;\;|\;\;C\right]}_{R+1}$$
  (11) $${\bar{X}}^{C}=[\bar{X}\;\;|\;\;\ldots \;\;|\;\;\bar{X}{]}_{R+1}$$
  By doing this, we can compute the Hadamard product between $C^{C}$ and ${\bar{X}}^{C}$ in Step 7, and then subtract the result from X in Step 8, in a direct way.
- Step 7: Applying the weights $C^{C}$ on the tensor ${\bar{X}}^{C}$.
  Next, in Step 7 of Figure 1, we compute the Hadamard product between $C^{C}$ and ${\bar{X}}^{C}$ such that the weights computed in Step 5 are applied to each element of the average common feature tensor, i.e.,
  (12) $$W=C^{C}⊙{\bar{X}}^{C}$$
- Step 8: Computing the filtered dataset tensor, $X^{\left[f\right]}$.
  Then, in Step 8 of Figure 1, the filtered dataset tensor $X^{\left[f\right]}\in R^{N_{1}\times \cdots \times N_{R}\times M}$ can be computed as follows:
  (13) $$X^{\left[f\right]}=X-W$$
- Step 9: Obtaining the (R+1)-th mode unfolding matrix, ${\left[X\right]}_{\left(R+1\right)}^{\left[f\right]}$.
  Finally, in Step 9 of Figure 1, we obtain the (R+1)-th mode unfolding matrix of $X^{\left[f\right]}$, given by ${\left[X\right]}_{\left(R+1\right)}^{\left[f\right]}\in R^{M\times N}$. Similarly to Equation (6), each element $\left(x_{r}^{\left[f\right]},j\right)$ of the *r*-th unfolding matrix ${\left[X\right]}_{r}^{\left[f\right]}$ is computed as follows:
  (14) $$j=1+\sum_{\begin{array}{c} k=1\\ k\neq r \end{array}}^{R+1}\left(x_{k}^{\left[f\right]}-1\right)J_{k},\;\;\mathrm{with}\;\;J_{k}=\prod_{\begin{array}{c} m=1\\ m\neq r \end{array}}^{k-1}N_{m}$$
  Such a matrix is forwarded to the ML classification algorithm for classification tasks, where the predicted class label vector $\hat{y}\in R^{M}$ is computed. Since decision tree, random forest and gradient boosting algorithms present considerable results in network intrusion detection problems, they are adopted in this paper for classifying the network traffic data [14].

The proposed average common feature extraction technique for DDoS attack detection in CPSs is summarized in Algorithm 1.
**Algorithm 1:** Proposed average common feature extraction technique for DDoS attack detection.**Input**: - Dataset tensor $X\in R^{N_{1}\times \cdots \times N_{R}\times M}$ - Multilinear rank $\left(d_{1},\ldots ,d_{R+1}\right)$ **Output**: - Filtered dataset matrix ${\left[X\right]}_{\left(R+1\right)}^{\left[f\right]}\in R^{M\times N}$ **Algorithm Steps:** 1 Compute the HOSVD of $X\in R^{N_{1}\times \cdots \times N_{R}\times M}$, with multilinear rank $\left(d_{1},\ldots ,d_{R+1}\right)$, as in (3) 2 Compute the common feature tensor $\tilde{X}\in R^{N_{1}\times \cdots \times N_{R}\times d_{R+1}}$ as in (4) 3 Compute the average common feature tensor $\bar{X}\in R^{N_{1}\times \cdots \times N_{R}}$ as in (5) 4 Convert X into the (R+1)-th mode unfolding matrix ${\left[X\right]}_{\left(R+1\right)}\in R^{M\times N}$ as in (6) 5 Obtain the weight tensor $C\in R^{N_{1}\times \cdots \times N_{R}}$, whose elements are computed as in (7) to (9) 6 Obtain the concatenated tensors $C^{C}\in R^{N_{1}\times \cdots \times N_{R}\times M}$ and ${\bar{X}}^{C}\in R^{N_{1}\times \cdots \times N_{R}\times M}$ as in (10) and (11) 7 Compute the Hadamard product between $C^{C}$ and ${\bar{X}}^{C}$ as in (12) 8 Compute the filtered dataset tensor $X^{\left[f\right]}\in R^{N_{1}\times \cdots \times N_{R}\times M}$ as in (13) 9 Convert $X^{\left[f\right]}$ into the (R+1)-th mode unfolding matrix ${\left[X\right]}_{\left(R+1\right)}^{\left[f\right]}\in R^{M\times N}$ as in (14)

## 6. Simulation Results

This section presents the simulation results and is divided into four subsections. Section 6.1 and Section 6.2 introduce and discuss the results obtained from numerical simulations, respectively. Next, the comparison between the proposed technique and related works is shown in Section 6.3. Finally, Section 6.4 presents the computational complexity of the compared schemes.

### 6.1. Results

In this paper, we adopt Accuracy, Detection Rate, False Alarm Rate, Area Under the Precision–Recall Curve and Matthews Correlation Coefficient as performance evaluation metrics. Furthermore, the Relative Loss of Accuracy is adopted as error-robustness evaluation metric. Such metrics are based on the values of true positives (TP), true negatives (TN), false positives (FP) and false negatives (FN). TP and TN represent the correctly predicted values, whereas FP and FN correspond to the misclassified events. These metrics are defined as follows:

- Accuracy (Acc): the ratio between the correctly predicted instances and the total number of instances,
  (15) $$\mathrm{Acc}=\frac{\mathrm{TP}+\mathrm{TN}}{\mathrm{TP}+\mathrm{TN}+\mathrm{FP}+\mathrm{FN}}$$
- Detection Rate (DR): the ratio between the correctly predicted positive instances and the total number of actual positive instances,
  (16) $$\mathrm{DR}=\frac{\mathrm{TP}}{\mathrm{TP}+\mathrm{FN}}$$
- False Alarm Rate (FAR): the ratio between the number of negative instances wrongly classified as positives and the total number of actual negative instances,
  (17) $$\mathrm{FAR}=\frac{\mathrm{FP}}{\mathrm{TN}+\mathrm{FP}}$$
- Area Under the Precision–Recall Curve (AUPRC): reflects a trade-off between the precision and recall. Precision is the ability of a classifier not to label as positive a sample that is negative, defined as Prec=TP/(TP+FP). On the other hand, recall corresponds to the ability of a classifier to find all positive samples, given by Rec=TP/(TP+FN). The AUPRC corresponds to the area under the curve obtained by plotting the precision and recall on the y and x axes, respectively, for different probability thresholds. By applying the trapezoidal rule, the AUPRC can be defined as:
  (18) $$\mathrm{AUPRC}=\frac{1}{2}\sum_{k=2}^{K}\left({\mathrm{Prec}}_{k}+{\mathrm{Prec}}_{k-1}\right)\cdot \left({\mathrm{Rec}}_{k}-{\mathrm{Rec}}_{k-1}\right)$$
  where ${\mathrm{Prec}}_{k}$ and ${\mathrm{Rec}}_{k}$ are the precision and recall values for the *k*-th threshold, and *K* is the total number of probability thresholds.
  Matthews Correlation Coefficient (MCC): measures the quality of binary classifications. It ranges from −1 to +1 such that higher values represent better performance. The MCC is defined as:
  (19) $$\mathrm{MCC}=\frac{\left(\mathrm{TP}\cdot \mathrm{TN})-(\mathrm{FP}\cdot \mathrm{FN}\right)}{\sqrt{\left(\mathrm{TP}+\mathrm{FP})\cdot (\mathrm{TP}+\mathrm{FN})\cdot (\mathrm{TN}+\mathrm{FP})\cdot (\mathrm{TN}+\mathrm{FN}\right)}}$$
- Relative Loss of Accuracy (RLA): measures the percentage of variation of the accuracy of the classifiers at the error level EL%, ${\mathrm{Acc}}_{\mathrm{EL}\%}$, with respect to the original case with no additional error, ${\mathrm{Acc}}_{0\%}$,
  (20) $$\mathrm{RLA}=\frac{{\mathrm{Acc}}_{0\%}-{\mathrm{Acc}}_{\mathrm{EL}\%}}{{\mathrm{Acc}}_{0\%}}$$

All experiments were executed on a desktop computer with processor Intel Core i7-2600 3.40 GHz and 16 GB of RAM. Data pre-processing and machine learning classifier algorithms were implemented in the Python library Scikit-Learn, whereas Python libraries Tensorly [27] and HOTTBOX [26] were used to implement tensor computations. Furthermore, the proposed approach is validated by considering subsets of the CICDDoS2019 and CICIDS2017 datasets, described in Section 4.2. A total of *M* = 40,000 instances were extracted from each dataset, of which 20% correspond to DDoS attacks, as detailed in Table 3.

CICDDoS2019 is a novel dataset that contains an extensive variety of DDoS attacks and fills the gaps of the current datasets [28]. In this sense, it is used for performance evaluation throughout this subsection. The proposed scheme is compared with state-of-the-art low-rank approximation techniques, namely, the Higher-Order Orthogonal Iteration (HOOI) [12] and Higher-Order Singular Value Decomposition (HOSVD) [11]. Here, we intend to assess the performance of the proposed approach in the presence of corrupted datasets, as well as its error-robustness.

The dataset is folded as a three-dimensional tensor with size $N_{1}\times N_{2}\times M$, i.e., R+1=3. For simplicity, we set $N_{1}=N_{2}=8$ such that the number of features is given by $N_{1}\cdot N_{2}=N=64$. The dataset is split into training, validation and testing sets, with proportion 60:20:20. The validation set is used for hyperparameter tuning, whereas the testing set is used only once for performance evaluation. In addition, we also evaluate the proposed technique for different training dataset sizes.

In accordance with the literature about corrupted datasets [8], we adopt the following error generation process: for each feature $X_{:,n}$ for n=1,…,N, EL % of the instances are corrupted with Gaussian noise with mean zero and standard deviation $(\max\left(X_{:,n}\right)-\min\left(X_{:,n}\right))/5$. We simulate a total of 100 different experiments, using the decision tree (DT), random forest (RF) and gradient boosting (GB) as machine learning classifiers. The *R*-D MDL scheme [25] is applied to estimate the multilinear rank of the training and testing datasets.

Table 4 shows the accuracy, detection rate, false alarm rate, area under the precision–recall curve and Matthews correlation coefficient as a function of the error level (EL). The EL ranges from 10% to 30%. For each error level and ML classifier, the best metric values are highlighted in bold. From the results shown in Table 4, it is clear that the proposed scheme outperforms its competitor methods for all EL range. In addition, even in high error level conditions, e.g., EL = 30%, the proposed technique presents outstanding results, with Acc = 98.94%, DR = 97.70%, FAR = 4.35%, AUPRC = 0.9937 and MCC = 0.9663 when the random forest algorithm is applied for classification. Furthermore, we observe that the AUPRC is higher than 0.98 for all EL range when using RF and GB classifiers, which reflects a considerable trade-off between the true positive rate and positive predictive values. Therefore, from Table 4, we note that the proposed technique presents a considerable performance along the whole error level range.

Next, the proposed approach is compared with the HOOI and HOSVD schemes when the training size proportion (TSP) ranges from 20% to 70% of all available instances, with error-level fixed in 20%. Table 5 shows the Acc, DR, FAR, AUPRC and MCC for different values of TSP. For each training size proportion and ML classifier, we highlight in bold the best metric values that were obtained. It can be observed that the proposed scheme delivers significantly better results when compared to its competitor methods in all TSP range, showing outstanding metric values. Note that, even with small training datasets, e.g., TSP = 20%, the proposed approach presents Acc, DR, FAR, AUPRC and MCC equal to 99.18%, 98.85%, 1.71%, 0.9976 and 0.9746, respectively, when RF is applied for classification. Therefore, our proposed approach shows considerable performance, even when trained with small data.

**Figure 2:**
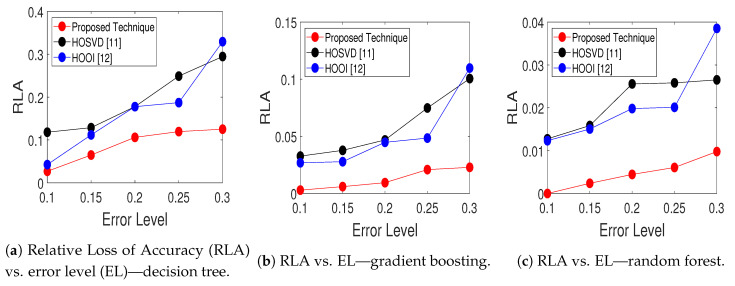
Plots of relative loss of accuracy, as a function of the error level, for the following machine learning (ML) classifiers: (**a**) decision tree, (**b**) gradient boosting, (**c**) random forest.

Finally, the error-robustness evaluation results of the proposed scheme as well as the HOOI and HOSVD approaches are presented. The same simulation parameters adopted in the experiments of Table 4 are considered. Figure 2 illustrates the relative loss of accuracy, as a function of the error level, for each compared technique and different ML classifiers. As expected, all techniques presented an improved performance for lower error levels, in which the datasets present lower corruption. Furthermore, note that the proposed approach shows outstanding metric values for all EL range. As shown in Figure 2, the RLA is approximately zero when the error level is 10%, and is lower than 12% for EL = 30%, regardless of the classifier. In this sense, it can be seen that the proposed approach shows a considerable error-robustness when compared to HOSVD and HOOI low-rank approximation techniques.

### 6.2. Discussion

In this paper, we compare the proposed technique with architectures in which the HOSVD and HOOI schemes are previously applied to the dataset tensor $X\in R^{N_{1}\times \cdots \times N_{R}\times M}$ for denoising. The HOSVD is a generalization of the matrix Singular Value Decomposition to higher-order tensors and is widely applied for noise reduction. In this case, an (R+1)-th dimensional tensor X is decomposed into a core tensor and R+1 factor matrices truncated to the signal subspace, which is determined by the multilinear rank $\left(d_{1},\ldots ,d_{R+1}\right)$. On the other hand, HOOI is a low-rank approximation method in which more accurate truncated singular matrices and core tensor are computed through higher order orthogonal iterations.

Decision tree, random forest and gradient boosting are adopted as ML classifiers. Despite its low computational cost and ease of understanding and interpretation, decision tree presents high variance, i.e., completely different trees can be generated from tiny changes in the training dataset. When trained with corrupted data or small datasets, DTs can lead to overfitting. Such fact can be seen in Table 4 and Table 5, in which DTs are outperformed by both GB and RF, especially for high error levels and small TSP. For instance, in Table 4, for EL = 25%, the proposed technique presents values of MCC for DT, GB and RF classifiers equal to 62.68%, 97.68% and 97.81%, respectively. As expected, all compared techniques deliver better performance when RF and GB are used for classification, since both algorithms reduce the variance existing in DTs and prevent overfitting. Random forest and gradient boosting combine multiple DTs, but with different tree-building processes: while the former builds each tree independently and combines results at the end, the latter builds one tree at a time, combining results during the process.

Furthermore, from Table 4, it can be observed that, for all compared schemes, RF outperforms GB for almost all EL range. Since gradient boosting combines the results along the process, it is more sensitive to data corruption, resulting in overfitting. For example, for EL = 30%, the values of DR for random forest and gradient boosting when considering our proposed approach are, respectively, 97.70% and 96.02%. Such a fact is more evident in HOSVD, which presents detection rates of 92.67% and 85.27% for RF and GB, respectively. In addition, from the results shown in Table 4, note that the proposed scheme outperforms both HOSVD and HOOI techniques for all EL range. Such results confirm that ML classifiers benefit from the more discriminative individual information resulting from the average common feature extraction technique applied on the training dataset. Note that HOSVD is also outperformed by HOOI for high error levels, confirming that the latter scheme leads to better results due to the more accurate core tensor and singular matrices generated through alternating least squares decomposition methods. In short, the compared techniques present better performance as the error level is lower. In this case, the machine learning classifiers deal with less corrupted data and, consequently, deliver more reliable and accurate results.

Additionally, from Table 5, we observe that, in general, the compared techniques present better performance as the training size proportion is higher. Small training datasets can lead to a lack of representative instances and, consequently, to overfitting. In this case, the ML algorithm is excessively adjusted to the training data, performing poorly in predicting new instances. As mentioned above, such a fact is more evident in decision trees, which are more prone to overfitting. For instance, when considering the smallest training dataset size, i.e., TSP = 20%, the values of AUPRC for the proposed scheme, HOSVD and HOOI when DT is applied for classification are, respectively, 0.7804, 0.7437 and 0.6099. On the other hand, the proposed approach is very robust against small training dataset sizes when gradient boosting and random forests are used for classification. For example, still considering the worst case of TSP = 20%, the AUPRC for the proposed scheme when GB and RF are applied are, respectively, 0.9953 and 0.9976. However, both HOSVD and HOOI present a performance reduction in this case, showing AUPRC of 0.8168 and 0.9336 for gradient boosting, and 0.9845 and 0.9869 for the random forest, respectively.

Finally, the error-robustness of all compared approaches is assessed in Figure 2, in which the relative loss of accuracy is illustrated. By observing Figure 2c, we observe that all schemes are more robust against errors for random forest classifier when compared to DT and GB algorithms. For instance, considering the worst case of EL = 30%, the proposed technique presents RLA of 0.98% when RF is applied for classification. On the other hand, for GB and DT, our approach shows relative loss of accuracy of 2.29% and 12.52%, respectively. Therefore, once again we observe that random forest outperforms the DT and GB algorithms in DDoS attack detection.

### 6.3. Performance Comparison with Related Works

This subsection presents the performance comparison between the proposed scheme and related works assuming error-free conditions. Furthermore, since CICIDS2017 has been extensively applied for IDS validation by several papers in the literature, we also include the performance evaluation on such dataset. Consequently, the comparison with related researches is enriched due to the higher number of competing schemes.

Since the related papers assume error-free datasets, the proposed approach is considered with error level 0% for comparison. Table 6 shows the adopted dataset, the ML classification algorithm and the values of accuracy, detection rate and false alarm rate obtained by the proposed approach and the related papers. The metrics represented as "Not Available" (N/A) were not informed by the corresponding paper. Furthermore, since CICDDoS2019 is a new released dataset, to the best of our knowledge only Elsayed et al. [28] applied such data for performance evaluation. The authors proposed a deep learning-based intrusion detection system in which a recurrent neural network is combined with an autoencoder. Note that, considering the CICDDoS2019 dataset, the proposed technique outperforms the competing scheme when GB and RF algorithms are applied for classification. Our approach presents Acc = 99.87% and DR = 99.86% for gradient boosting, whereas accuracy and detection rate of 99.55% and 98.96% were obtained when using random forest classifier.

On the other hand, as above mentioned, CICIDS2017 was applied by several authors for IDS performance evaluation, as it can be seen in Table 6. Although it is not the best IDS among the compared ones, the proposed scheme still presents a considerable performance, with Acc = 99.95%, DR = 99.95% and FAR = 0.05% for gradient boosting algorithm, outperforming almost all competitor schemes. It is worth to mention the performance shown by LUCID, proposed by Doriguzzi-Corin et al. in [29], with Acc, DR and FAR of 99.67%, 99.94% and 0.59%, respectively. The authors presented a practical, lightweight CNN-based DDoS detection architecture with low processing overhead and attack detection time. In addition, the 1D-CNN-LSTM model, proposed by Roopak et al. [30], showed a considerable detection rate of 99.10%. Note that both papers propose deep learning-based schemes, which usually deliver better performance when compared to traditional machine learning-based solutions, such as the DT, RF and GB algorithms.

### 6.4. Computational Complexity

This section discusses the computational complexity of the proposed approach, described in Algorithm 1. For simplicity, the complexity is analyzed for a three-dimensional dataset tensor $X\in R^{N_{1}\times N_{2}\times M}$. We only consider the most costly calculations, represented by Steps 1 to 3 of Algorithm 1, as a function of the most important variables, namely, $N_{1}$, $N_{2}$, *M* and $\left(d_{1},d_{2},d_{3}\right)$. Consequently, the computational cost related to folding and unfolding of matrices and tensors, performed in Steps 4 and 9 of Algorithm 1, are not considered since such functions are about data representations. Similarly, the time complexity of the Steps 5–8 of Algorithm 1 are not analyzed, since low-cost computations are performed in such steps.

Step 1 of Algorithm 1 corresponds to the HOSVD of the dataset tensor X and presents computational complexity given by [33]:

(21) $$O\left[\mathrm{HOSVD}\right]=O\left[\sum_{j=1}^{3}\left(N_{j}\prod_{k=1}^{3}N_{k}\right)+\sum_{j=1}^{3}\left(\prod_{k=1}^{j}d_{k}\prod_{k=j}^{3}N_{k}\right)\right]$$

where, for simplicity of notation, $N_{3}$ corresponds to the number of dataset instances *M*.

Next, in Steps 2 and 3 of Algorithm 1, we compute the common feature tensor as well as its average along the 3-rd dimension. Such steps require two tensor times matrix products plus the average calculation, and present complexity given by:

(22) $$O\left[\mathrm{CF}\right]=O\left[N_{1}^{2}N_{2}d_{3}\right]+O\left[N_{1}N_{2}^{2}d_{3}\right]+O\left[N_{1}N_{2}d_{3}\right]$$

Finally, the overall computational complexity of Algorithm 1 corresponds to the sum of the above mentioned complexities,

(23) O[Final]=O[HOSVD]+O[CF]

In Table 7, we summarize the computational complexities of the proposed approach as well as the HOOI and HOSVD techniques. For HOOI, *I* corresponds to the number of iterations and $d=\max(d_{1},d_{2},d_{3})$. Note that the proposed scheme is accompanied by an increase of computational complexity, which reinforces the trade-off between the more accurate DDoS attack detection and the time cost.

## 7. Conclusions

In this paper, we propose a novel average common feature extraction technique applied on DDoS attack detection. Initially, the proposed scheme filter out, from the dataset, the average value of the common features among instances by applying the classic Higher-Order Singular Value Decomposition. Finally, the filtered dataset is sent to machine learning algorithms where data are classified as benign traffic or DDoS attack.

Extensive numerical simulations are performed on the CICDDoS2019 and CICIDS2017 benchmark datasets, whereas decision tree, random forest and gradient boosting are used as ML classifiers. Further, accuracy, detection rate, false alarm rate, area under the precision–recall curve, Matthews correlation coefficient and relative loss of accuracy are adopted as evaluation metrics. According to the obtained results, the proposed scheme outperforms the traditional HOSVD and HOOI techniques, presenting a higher error-robustness. For instance, considering a dataset corruption level of 30%, the proposed scheme shows values of Acc, DR, FAR, AUPRC and MCC of 98.94%, 97.70%, 4.35%, 0.9937 and 0.9663, respectively, when random forest algorithm is used for classification. In the same conditions, the traditional HOOI technique shows Acc, DR, FAR, AUPRC and MCC equal to 97.65%, 94.19%, 11.52%, 0.9906 and 0.9250, respectively. In addition, we observe that our proposed scheme presents high robustness against small training datasets, showing a slight loss of performance along the whole evaluated TSP. For example, when the training dataset size is only 20% of all available samples, the proposed approach shows Acc, DR, FAR, AUPRC and MCC equal to 99.18%, 98.85%, 1.71%, 0.9976 and 0.9746, respectively, for random forest classifier. On the other hand, considering the same TSP, the well-known HOSVD scheme presents values of Acc, DR, FAR, AUPRC and MCC of 97.55%, 95.20%, 8.71%, 0.9845 and 0.9227, respectively. However, an important drawback of our proposed scheme is its higher computational complexity, which reflects the trade-off between the more accurate DDoS attack detection and the time cost.

Another considerable finding corresponds to the performance of the evaluated ML classification algorithms for DDoS attack detection. According to simulations, decision trees are more prone to overfitting when data are highly corrupted or small datasets are used for training. For example, for a data corruption level of 25%, the proposed technique presents a detection rate of 80.23% when DTs are used for classification, whereas 98.57% and 98.66% are obtained with GB and RF, respectively. Similarly, for a training dataset size proportion of 30%, our approach obtained accuracies of 98.55% and 98.95% with GB and RF algorithms, while Acc = 85.95% when decision tree is applied. Additionally, it is observed that the random forest classifier presents higher error-robustness when compared to gradient boosting. For instance, considering a data corruption level of 20%, our proposed scheme shows a relative loss of accuracy of 0.61% when RF is applied for classification, whereas 2.09% is obtained for GB. Therefore, it is shown that gradient boosting is more sensitive to data corruption when compared to random forest, since the former scheme builds one tree at a time and combines results along the process, whereas the latter builds each tree independently, combining results at the end.

In the future, we intend to apply the proposed technique by using alternative machine learning algorithms, especially deep learning-based approaches, such as convolutional neural networks. Furthermore, we shall verify the performance of the proposed scheme for online DDoS attack detection.

## Figures and Tables

**Table 1:** Related works.

**Works related to multilinear algebra**				
**Paper**	**Aim**	**Proposed Solution**	**Pros**	**Cons**
Kisil et al. [10]	- Image classification.	- Common and individual feature extraction technique based on LL1 tensor decomposition.	- Flexible - Not restricted to images of the same dimensions. - Tensor-based solution.	- High computational complexity. - Corrupted datasets are not considered.
Rajwade et al. [11]	- Image denoising and classification.	- Patch-based ML technique for image denoising by applying HOSVD.	- Outstanding performance on grayscale and color images. - Tensor-based solution.	- Limited denoising performance.
Lathauwer et al. [12]	- Estimation of the best rank- $\left(R_{1},\ldots ,R_{N}\right)$ approximation of tensors.	- HOOI low-rank approximation algorithm.	- Outperforms HOSVD in the estimation of singular matrices and core tensor. - Tensor-based solution.	- High computational complexity.
**Works related to DDoS attack detection**				
**Paper**	**Aim**	**Proposed Solution**	**Pros**	**Cons**
Vieira et al. [5]	- Detection and identification of network attacks, including DDoS.	- Framework for detecting and identifying network attacks using model order selection, eigenvalues and similarity analysis.	- Outstanding accuracy for timely detection and identification of TCP and UDP ports under attack.	- Corrupted datasets are not considered. - Not based on ML techniques.
Hosseini and Azizi [13]	- DDoS attack detection.	- Hybrid framework based on data stream approach for detecting DDoS attacks.	- Computational process divided between client and proxy. - Early attack detection.	- Corrupted datasets are not considered.
Lima Filho et al. [14]	- DDoS attack detection.	- RF based DDoS detection system for early identification of TCP flood, UDP flood and HTTP flood.	- Early identification of volumetric attacks. - Packet inspection is not required.	- Corrupted datasets are not considered.
Wang et al. [6]	- DDoS attack detection.	- Feature selection combined with MLP. - Feedback mechanism to reconstruct the IDS according to detection errors.	- Feedback mechanism perceives errors based on recent detection results.	- Global optimal features are not necessarily found. - Corrupted datasets are not considered.

**Table 2:** Mathematical symbols along this paper.

Symbol	Definition	Symbol	Definition
X	Dataset matrix	G	Core tensor
$X_{0}$	Error-free dataset matrix	$\tilde{X}$	Common feature tensor
N	Error matrix	$\bar{X}$	Average common feature tensor
$X_{m,:}$	*m*-th dataset instance	C	Weight tensor
$X_{:,n}$	*n*-th dataset feature	$X^{\left[f\right]}$	Filtered dataset tensor
$A_{r}$	*r*-th factor matrix	${\left[X\right]}_{\left(r\right)}$	*r*-th mode unfolding matrix of X
$R_{\mathrm{xx}}$	Covariance matrix	$y_{m}$	Class label of $X_{m,:}$
E	Eigenvector matrix	*M*	Number of instances
Λ	Eigenvalue matrix	$M^{\mathrm{tr}}$	Number of training instances
y	Class label vector	$M^{\mathrm{te}}$	Number of testing instances
$\hat{y}$	Predicted class label vector	*N*	Number of features
X	Dataset tensor	$N_{r}$	Number of features along the *r*-th dimension
$X^{\mathrm{tr}}$	Training dataset tensor	R+1	Order of X
$X^{\mathrm{te}}$	Testing dataset tensor	$\left(d_{1}^{\mathrm{tr}},\ldots ,d_{R+1}^{\mathrm{tr}}\right)$	Multilinear rank of $X^{\mathrm{tr}}$
$X_{0}$	Error-free dataset tensor	$\left(d_{1}^{\mathrm{te}},\ldots ,d_{R+1}^{\mathrm{te}}\right)$	Multilinear rank of $X^{\mathrm{te}}$
N	Error tensor	$\left(\lambda_{1},\ldots ,\lambda_{N}\right)$	Eigenvalues of $R_{\mathrm{xx}}$

**Table 3:** DDoS attack types and the corresponding number of instances for each dataset.

Dataset	Traffic File	Traffic Type	Total
		Legitimate	32,000
		DNS-based DDoS	800
		LDAP-based DDoS	800
		MSSQL-based DDoS	800
		NetBIOS-based DDoS	800
CICDDoS2019	12 January 2019	NTP-based DDoS	800
		SNMP-based DDoS	800
		SSDP-based DDoS	800
		UDP flood	800
		TCP SYN flood	800
		TFTP-based DDoS	800
CICIDS2017	3 July 2017	Legitimate	32,000
	7 July 2017	DDoS LOIC	8000

**Table 4:** Performance evaluation for different error levels.

**EL**	**Model**	**Acc**			**FAR**			**MCC**			**AUPRC**			**DR**		
		**DT**	**GB**	**RF**	**DT**	**GB**	**RF**	**DT**	**GB**	**RF**	**DT**	**GB**	**RF**	**DT**	**GB**	**RF**
	Proposed	**0.9492**	**0.9958**	**0.9959**	**0.1308**	**0.0172**	**0.0147**	**0.8407**	**0.9866**	**0.9871**	**0.8855**	**0.9983**	**0.9975**	**0.9188**	**0.9909**	**0.9919**
**10%**	HOSVD [11]	0.8605	0.9659	0.9839	0.1701	0.0949	0.0766	0.6311	0.8922	0.9485	0.7405	0.9484	0.9908	0.8490	0.9429	0.9611
	HOOI [12]	0.9343	0.9707	0.9843	0.1313	0.0587	0.0722	0.7996	0.9098	0.9499	0.8542	0.9395	0.9946	0.9097	0.9596	0.9630
**EL**	**Model**	**Acc**			**FAR**			**MCC**			**AUPRC**			**DR**		
		**DT**	**GB**	**RF**	**DT**	**GB**	**RF**	**DT**	**GB**	**RF**	**DT**	**GB**	**RF**	**DT**	**GB**	**RF**
	Proposed	**0.9121**	**0.9892**	**0.9857**	**0.2201**	**0.0294**	**0.0681**	**0.7272**	**0.9666**	**0.9545**	**0.8076**	**0.9944**	**0.9974**	**0.8585**	**0.9822**	**0.9654**
**15%**	HOSVD [11]	0.8501	0.9608	0.9808	0.2916	0.0808	0.0925	0.5606	0.8820	0.9386	0.6855	0.9368	0.9902	0.7966	0.9451	0.9531
	HOOI [12]	0.8666	0.9501	0.9768	0.2324	0.1464	0.1049	0.6195	0.8405	0.9257	0.7279	0.8491	0.9837	0.8331	0.9137	0.9460
**EL**	**Model**	**Acc**			**FAR**			**MCC**			**AUPRC**			**DR**		
		**DT**	**GB**	**RF**	**DT**	**GB**	**RF**	**DT**	**GB**	**RF**	**DT**	**GB**	**RF**	**DT**	**GB**	**RF**
	Proposed	**0.9039**	**0.9844**	**0.9929**	**0.2398**	**0.0695**	**0.0284**	**0.6986**	**0.9502**	**0.9774**	**0.7829**	**0.9937**	**0.9966**	**0.8496**	**0.9640**	**0.9849**
**20%**	HOSVD [11]	0.8023	0.9517	0.9708	0.5040	0.2231	0.1348	0.3932	0.8427	0.9063	0.5699	0.9665	0.9795	0.6867	0.8858	0.9310
	HOOI [12]	0.6543	0.9538	0.9582	0.4227	0.1544	0.0930	0.2264	0.8521	0.8700	0.5019	0.9176	0.9617	0.6252	0.9130	0.9389
**EL**	**Model**	**Acc**			**FAR**			**MCC**			**AUPRC**			**DR**		
		**DT**	**GB**	**RF**	**DT**	**GB**	**RF**	**DT**	**GB**	**RF**	**DT**	**GB**	**RF**	**DT**	**GB**	**RF**
	Proposed	**0.8719**	**0.9927**	**0.9931**	**0.3125**	**0.0258**	**0.0240**	**0.6268**	**0.9768**	**0.9781**	**0.7413**	**0.9954**	**0.9966**	**0.8023**	**0.9857**	**0.9866**
**25%**	HOSVD [11]	0.6882	0.8981	0.9711	0.6180	0.1365	0.0942	0.1280	0.7245	0.9083	0.3906	0.7081	0.9722	0.5726	0.8850	0.9465
	HOOI [12]	0.8023	0.8889	0.9816	0.4281	0.3198	0.0857	0.4198	0.6585	0.9412	0.5884	0.7804	0.9877	0.7154	0.8102	0.9562
**EL**	**Model**	**Acc**			**FAR**			**MCC**			**AUPRC**			**DR**		
		**DT**	**GB**	**RF**	**DT**	**GB**	**RF**	**DT**	**GB**	**RF**	**DT**	**GB**	**RF**	**DT**	**GB**	**RF**
	Proposed	**0.8532**	**0.9759**	**0.9894**	**0.1782**	**0.0655**	**0.0435**	**0.6179**	**0.9266**	**0.9663**	**0.7335**	**0.9801**	**0.9937**	**0.8414**	**0.9602**	**0.9770**
**30%**	HOSVD [11]	0.7328	0.9238	0.9701	0.5221	0.2647	0.1449	0.2554	0.7496	0.9042	0.4796	0.8675	0.9878	0.6366	0.8527	0.9267
	HOOI [12]	0.7932	0.9717	0.9765	0.6998	0.0868	0.1152	0.2765	0.9102	0.9250	0.4818	0.9287	0.9906	0.6072	0.9496	0.9419

**Table 5:** Performance evaluation for different training size proportion, for a error level of 20%.

**TSP**	**Model**	**Acc**			**FAR**			**MCC**			**AUPRC**			**DR**		
		**DT**	**GB**	**RF**	**DT**	**GB**	**RF**	**DT**	**GB**	**RF**	**DT**	**GB**	**RF**	**DT**	**GB**	**RF**
	Proposed	0.8267	**0.9890**	**0.9918**	**0.1938**	**0.0457**	**0.0171**	0.5935	**0.9654**	**0.9746**	**0.7804**	**0.9953**	**0.9976**	**0.8190**	**0.9760**	**0.9885**
**20%**	HOSVD [11]	**0.8833**	0.8868	0.9755	0.4785	0.2786	0.0871	**0.6108**	0.6514	0.9227	0.7437	0.8168	0.9845	0.7478	0.8249	0.9520
	HOOI [12]	0.7805	0.9360	0.9740	0.3457	0.1151	0.0422	0.4296	0.8115	0.9207	0.6099	0.9336	0.9869	0.7332	0.9168	0.9679
**TSP**	**Model**	**Acc**			**FAR**			**MCC**			**AUPRC**			**DR**		
		**DT**	**GB**	**RF**	**DT**	**GB**	**RF**	**DT**	**GB**	**RF**	**DT**	**GB**	**RF**	**DT**	**GB**	**RF**
	Proposed	**0.8595**	**0.9855**	**0.9895**	**0.0660**	**0.0660**	**0.0454**	**0.6336**	0.8405	**0.9671**	**0.7817**	**0.9915**	**0.9948**	**0.8399**	**0.9662**	**0.9764**
**30%**	HOSVD [11]	0.7831	0.9565	0.9703	0.2618	0.0856	0.1141	0.4664	**0.8709**	0.9057	0.6382	0.8804	0.9705	0.7663	0.9408	0.9387
	HOOI [12]	0.7596	0.9287	0.9558	0.4072	0.2352	0.2182	0.3550	0.7704	0.8592	0.5534	0.9072	0.9841	0.6971	0.8673	0.8906
**TSP**	**Model**	**Acc**			**FAR**			**MCC**			**AUPRC**			**DR**		
		**DT**	**GB**	**RF**	**DT**	**GB**	**RF**	**DT**	**GB**	**RF**	**DT**	**GB**	**RF**	**DT**	**GB**	**RF**
	Proposed	**0.8252**	**0.9847**	**0.9845**	**0.1977**	**0.0542**	**0.0494**	0.5860	**0.9518**	**0.9513**	**0.7743**	**0.9882**	**0.9922**	**0.8166**	**0.9701**	**0.9718**
**40%**	HOSVD [11]	0.7588	0.9170	0.9735	0.4338	0.0728	0.1194	0.3411	0.7769	0.9154	0.5416	0.9022	0.9801	0.6864	0.9209	0.9385
	HOOI [12]	0.5839	0.8913	0.9610	0.6249	0.2576	0.1760	**0.6635**	0.6638	0.8747	0.3484	0.8263	0.9575	0.5054	0.8353	0.9095
**TSP**	**Model**	**Acc**			**FAR**			**MCC**			**AUPRC**			**DR**		
		**DT**	**GB**	**RF**	**DT**	**GB**	**RF**	**DT**	**GB**	**RF**	**DT**	**GB**	**RF**	**DT**	**GB**	**RF**
	Proposed	**0.9026**	**0.9814**	**0.9903**	**0.2605**	**0.0784**	**0.0396**	**0.6963**	**0.9418**	**0.9696**	**0.8087**	**0.9663**	**0.9972**	**0.8417**	**0.9591**	**0.9791**
**50%**	HOSVD [11]	0.7211	0.9471	0.9834	0.4713	0.1930	0.0750	0.2729	0.8325	0.9479	0.5047	0.9435	0.9936	0.6493	0.8948	0.9615
	HOOI [12]	0.8395	0.9319	0.9698	0.3490	0.0844	0.0839	0.5238	0.8121	0.9060	0.6614	0.7812	0.9758	0.7691	0.9258	0.9498
**TSP**	**Model**	**Acc**			**FAR**			**MCC**			**AUPRC**			**DR**		
		**DT**	**GB**	**RF**	**DT**	**GB**	**RF**	**DT**	**GB**	**RF**	**DT**	**GB**	**RF**	**DT**	**GB**	**RF**
	Proposed	**0.9062**	**0.9815**	**0.9943**	**0.2103**	**0.0439**	**0.0209**	**0.7180**	**0.9420**	**0.9820**	**0.8523**	**0.9833**	**0.9972**	**0.8515**	**0.9587**	**0.9886**
**60%**	HOSVD [11]	0.8134	0.9623	0.9623	0.2432	0.0673	0.0543	0.5353	0.8898	0.8882	0.6820	0.9330	0.9781	0.8035	0.9571	0.9561
	HOOI [12]	0.8199	0.9251	0.9558	0.3815	0.2714	0.2154	0.4862	0.7651	0.8598	0.6417	0.8730	0.9838	0.7446	0.8516	0.8918
**TSP**	**Model**	**Acc**			**FAR**			**MCC**			**AUPRC**			**DR**		
		**DT**	**GB**	**RF**	**DT**	**GB**	**RF**	**DT**	**GB**	**RF**	**DT**	**GB**	**RF**	**DT**	**GB**	**RF**
	Proposed	**0.9065**	**0.9826**	**0.9937**	**0.2817**	**0.0427**	**0.0287**	**0.6928**	**0.9458**	**0.9800**	**0.8323**	**0.9658**	**0.9978**	**0.9277**	**0.9731**	**0.9853**
**70%**	HOSVD [11]	0.7527	0.8902	0.9578	0.1265	0.0490	0.0781	0.4885	0.7391	0.8708	0.6699	0.8573	0.9688	0.7982	0.9131	0.9443
	HOOI [12]	0.7727	0.9455	0.9599	0.2222	0.1584	0.0594	0.4668	0.8259	0.8791	0.6421	0.9407	0.9736	0.7746	0.9064	0.9526

**Table 6:** Comparison between the proposed technique and related papers.

Dataset	Paper	ML Algorithm	Acc	DR	FAR
	Proposed scheme	DT	0.9754	0.9509	0.0895
CICDDoS2019	Proposed scheme	GB	0.9987	0.9986	0.0016
	Proposed scheme	RF	0.9955	0.9896	0.0201
	Elsayed et al. [28]	RNN+AutoEncoder	0.9900	0.9900	N/A
	Proposed scheme	DT	0.9994	0.9993	0.0007
	Proposed scheme	GB	0.9995	0.9995	0.0005
	Proposed scheme	RF	0.9996	0.9989	0.0022
	Lopez et al. [31]	RF	0.9900	N/A	N/A
	Doriguzzi–Corin et al. [29]	LUCID	0.9967	0.9994	0.0059
CICIDS2017	Lima Filho et al. [14]	RF	N/A	0.8000	0.0020
	Aamir and Ali Zaidi [32]	RF	0.9666	N/A	N/A
	Roopak et al. [30]	MLP	0.8634	0.8625	N/A
	Roopak et al. [30]	1D-CNN	0.9514	0.9017	N/A
	Roopak et al. [30]	LSTM	0.9624	0.8989	N/A
	Roopak et al. [30]	1D-CNN+LSTM	0.9716	0.9910	N/A

**Table 7:** Time complexity for the proposed approach, as well as the HOSVD and HOOI low-rank approximation techniques.

Algorithm	Time Complexity
Proposed Technique	$O\left[\sum_{j=1}^{3}\left(N_{j}\prod_{k=1}^{3}N_{k}\right)+\sum_{j=1}^{3}\left(\prod_{k=1}^{j}d_{k}\prod_{k=j}^{3}N_{k}\right)\right]+$ $+O\left[N_{1}^{2}N_{2}d_{3}\right]+O\left[N_{1}N_{2}^{2}d_{3}\right]+O\left[N_{1}N_{2}d_{3}\right]$
HOSVD [11]	$O\left[\sum_{j=1}^{3}\left(N_{j}\prod_{k=1}^{3}N_{k}\right)+\sum_{j=1}^{3}\left(\prod_{k=1}^{j}d_{k}\prod_{k=j}^{3}N_{k}\right)\right]$
HOOI [12]	$O\left[M^{3}dI\right]+O\left[M^{2}d^{2}I\right]+O\left[M^{3}d\right]+O\left[Md^{3}\right]$

## Abbreviations

The following abbreviations are used in this manuscript:

Acc	Accuracy
AUPRC	Area Under the Precision-Recall Curve
CIC	Canadian Institute for Cybersecurity
CNN	Convolutional Neural Network
CPS	Cyber-Physical System
CPU	Central Processing Unit
DDoS	Distributed Denial of Service
DoS	Denial of Service
DNS	Domain Name System
DR	Detection Rate
DT	Decision Tree
FAR	False Alarm Rate
GB	Gradient Boosting
HOOI	Higher Order Orthogonal Iteration
HOSVD	Higher Order Singular Value Decomposition
HTTP	HyperText Transfer Protocol
ICMP	Internet Control Message Protocol
IDS	Intrusion Detection System
IoT	Internet of Things
IP	Internet Protocol
LDAP	Lightweight Directory Access Protocol
LSTM	Long Short Term Memory
MCC	Matthews Correlation Coefficient
MDL	Minimum Description Length
ML	Machine Learning
MOS	Model Order Selection
MSSQL	Microsoft Structured Query Language
NaN	Not a Number
NetBIOS	Network Basic Input/Output System
NTP	Network Time Protocol
N/A	Not Available
OSI	Open System Interconnection
R-D MDL	R-Dimensional Minimum Description Length
RF	Random Forest
RLA	Relative Loss of Accuracy
SNMP	Simple Network Management Protocol
SQL Injection	Structured Query Language Injection
SSDP	Simple Service Discovery Protocol
TCP	Transmission Control Protocol
TFTP	Trivial File Transfer Protocol
TSP	Training Size Proportion
UDP	User Datagram Protocol
XSS	Cross-Site Scripting
